# *Notes from the Field:* Increase in Reported Hepatitis A Infections Among Men Who Have Sex with Men — New York City, January–August 2017

**DOI:** 10.15585/mmwr.mm6637a7

**Published:** 2017-09-22

**Authors:** Julia Latash, Marie Dorsinville, Paula Del Rosso, Mike Antwi, Vasudha Reddy, HaeNa Waechter, Jacqueline Lawler, Heather Boss, Philip Kurpiel, P. Bryon Backenson, Charles Gonzalez, Shannon Rowe, Tasha Poissant, Yulin Lin, Guo-Liang Xia, Sharon Balter

**Affiliations:** ^1^Bureau of Communicable Disease, New York City Department of Health and Mental Hygiene; ^2^Council of State and Territorial Epidemiologists/CDC Applied Epidemiology Fellowship; ^3^Orange County Department of Health, New York; ^4^Metropolitan Area Regional Office, New York State Department of Health; ^5^Bureau of Communicable Disease Control, New York State Department of Health; ^6^El Paso County Public Health, Colorado; ^7^Acute & Communicable Disease Prevention Section, Oregon Health Authority; ^8^Division of Viral Hepatitis Laboratory, CDC.

Since 2011, the New York City (NYC) Department of Health and Mental Hygiene (DOHMH) has typically been notified of three or fewer cases of hepatitis A virus (HAV) infection each year among men who have sex with men (MSM) who reported no travel to countries where HAV is endemic. This year, DOHMH noted an increase in HAV infections among MSM with onsets in January–March 2017, and notified other public health jurisdictions via Epi-X, CDC’s communication exchange network. As a result, 51 patients with HAV infection involving MSM were linked to the increase in NYC.

Confirmed cases were defined as symptomatic HAV infections with onset after December 31, 2016, in NYC residents who reported being MSM or having sexual contact with MSM, and reported no travel to areas of high or intermediate HAV endemicity. Probable cases were defined as onset of symptomatic HAV infection after December 31, 2016, in NYC residents who, irrespective of travel, reported being MSM or having sexual contact with MSM. For the period January 1–August 31, 2017, DOHMH identified 46 cases in MSM or persons with sexual contact with MSM; 36 confirmed and nine probable cases occurred in 45 MSM patients and one was in a female (confirmed case) who reported sexual contact with a bisexual male resident of a New York county outside New York City. Fifteen (33%) of the 46 patients were hospitalized, and three (7%) reported previous receipt of hepatitis A vaccine. Nineteen (41%) patients had traveled domestically during their incubation period, and eight (17%) had traveled to Western European countries where outbreaks of HAV infection among MSM are ongoing (*1*).

NYC routine surveillance identified another case of HAV infection (in addition to the 46 NYC patients), in a man who was hospitalized in New York City but resided in the New York county that had been visited by the female patient. Several Colorado jurisdictions also contacted DOHMH to report increases in HAV infections among MSM. In total, 51 patients were linked to the increase in NYC, either through epidemiologic or laboratory evidence, including five non-NYC patients (three from Colorado, one from New York outside of NYC, and one from Oregon).

Three of the 46 NYC patients and the one patient from Oregon reported sexual contact with four NYC outbreak patients (Figure). The Oregon patient (illness onset March 2017) worked as a food handler at a restaurant in Oregon, and a second food handler in the establishment subsequently contracted HAV infection, prompting a public notification recommending postexposure prophylaxis for an estimated 1,000 patrons who ate or drank at the establishment during a 7-day period in March 2017.

**FIGURE:**
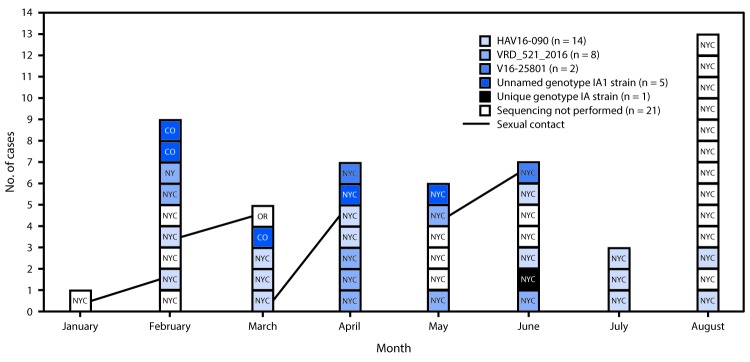
Number of reported cases of hepatitis A virus (HAV) infection involving men who have sex with men (N = 51), by state or city of residence, month of symptom onset, HAV genotype, and reported sexual contact — New York City, January–August, 2017 **Abbreviations:** CO = Colorado; NY = New York (non-NYC); NYC = New York City; OR = Oregon.

Serum specimens from 25 NYC MSM patients, the NYC female patient, and the New York (non-NYC) MSM patient were sent to CDC’s Division of Viral Hepatitis Laboratory for molecular sequencing. Sequences of HAV isolated from the serum of 24 patients, including four of the eight who had traveled to Europe, matched the strains of genotype IA HAV circulating among European MSM: HAV16–090 (14 patients), VRD_521_2016 (eight), V16–25801 (two); two patients had sequences matching three Colorado MSM patients, and one had a unique sequence (Figure).

Only three patients with HAV infection reported previous receipt of HAV vaccine; this ongoing investigation highlights the importance of HAV vaccination among MSM, and of determining MSM status during HAV investigations. One patient received 1 dose (as postexposure prophylaxis), but the doses for the other two patients were unknown; both reported previous receipt of HAV vaccine but did not know the number of doses. Since 1996, the Advisory Committee on Immunization Practices has recommended that all MSM receive 2 doses of HAV vaccine administered at least 6 months apart (*2*). In NYC, the incidence of HAV infection for 2013–2015 was 6.8 times higher among MSM adults who had not traveled to countries where HAV is endemic than among non-MSM adults.* HAV vaccine was added to the routine childhood immunization schedule in 2006, but many susceptible adults might still be unvaccinated. Efforts to promote HAV vaccine in MSM, including targeted messaging campaigns,^†^ will help prevent transmission among MSM (*2*).^§^

## Footnotes

* Incidence rates were calculated using a numerator of combined 2013–2015 case reports of symptomatic HAV infections among NYC adults aged ≥18 years who reported no travel to countries of intermediate or high HAV endemicity, and a denominator of combined-year 2013–2015 estimates of MSM and non-MSM NYC adults aged ≥18 years from NYC’s Community Health Survey (CHS), an annual cross-sectional telephone survey conducted by DOHMH. Rates were age-adjusted using direct standardization to the U.S. 2000 standard population. MSM status was determined based on patient or provider report for HAV case investigations, and by respondent report in the CHS.

† http://www1.nyc.gov/site/doh/health/health-topics/hepatitis-a.page?utm_source=Twitter&utm_campaign=HepA.

^ §^ https://www.cdc.gov/hepatitis/populations/stds.htm.
